# Hypertensive disorders during pregnancy and polycystic ovary syndrome are associated with child communication and social skills in a sex-specific and androgen-dependent manner

**DOI:** 10.3389/fendo.2022.1000732

**Published:** 2022-09-29

**Authors:** Morgan R. Firestein, Russell D. Romeo, Hailey Winstead, Danielle A. Goldman, William A. Grobman, David M. Haas, Samuel Parry, Uma M. Reddy, Robert M. Silver, Ronald J. Wapner, Frances A. Champagne

**Affiliations:** ^1^ Department of Psychiatry, Columbia University Irving Medical Center, New York, NY, United States; ^2^ Departments of Psychology and Neuroscience and Behavior, Barnard College of Columbia University, New York, NY, United States; ^3^ Harvard Medical School, Harvard University, Boston, MA, United States; ^4^ Interdepartmental Neuroscience Program, Yale School of Medicine, New Haven, CT, United States; ^5^ Department of Obstetrics and Gynecology, The Ohio State University College of Medicine, Columbus, OH, United States; ^6^ Department of Obstetrics and Gynecology, School of Medicine, Indiana University, Indianapolis, IN, United States; ^7^ Department of Obstetrics and Gynecology, University of Pennsylvania, Philadelphia, PA, United States; ^8^ Department of Obstetrics & Gynecology, Columbia University Irving Medical Center, New York, NY, United States; ^9^ Department of Obstetrics and Gynecology, University of Utah, Salt Lake City, UT, United States; ^10^ Department of Psychology, University of Texas at Austin, Austin, TX, United States

**Keywords:** hypertension, polycystic ovary syndrome (PCOS), pregnancy, autism (ASD), neurodevelopment and intellectual disabilities, testosterone (androgen)

## Abstract

Prenatal exposure to testosterone is implicated in the etiology of autism spectrum disorder (ASD). Hypertensive disorders of pregnancy and polycystic ovary syndrome are associated with both hyperandrogenism and increased risk for ASD. We examined whether increased maternal testosterone mediates the relationship between these hyperandrogenic disorders (HDs) during pregnancy and child communication and social skills. Maternal plasma was collected during the second trimester and parent-report measures of child communication and social skills were obtained at 4.5-6.5 years of age from 270 participants enrolled in the Nulliparous Pregnancy Outcomes Study: Monitoring Mothers-to-be (nuMoM2b). Our retrospective frequency-matched cohort study design identified 58 mothers with one or both of the HDs and 58 matched controls. Women diagnosed with an HD who carried a female had higher testosterone levels compared to those carrying a male (*t*(56) = -2.70, *p* = 0.01). Compared to females controls, females born to women with an HD had significantly higher scores on the Social Communication Questionnaire (*t*(114) = -2.82, *p* =0.01). Maternal testosterone partially mediated the relationship between a diagnosis of an HD and SCQ scores among females. These findings point to sex-specific associations of two HDs – hypertensive disorders of pregnancy and polycystic ovary syndrome – on child communication and social skills and a mediating effect of maternal testosterone during pregnancy. Further research is needed to understand placental-mediated effects of maternal testosterone on child brain development and neurodevelopmental outcomes.

## Introduction

The increasing prevalence of autism spectrum disorder (ASD) has resulted in a growing number of theories focused on prenatal etiological influences. Elevated testosterone in amniotic fluid during critical periods of brain development have been implicated in risk for ASD (1). Associations have been observed between the concentration of testosterone in amniotic fluid and a range of subclinical ASD-related traits in early childhood including altered play behavior, reduced eye contact and vocabulary size, poorer emotion recognition, poorer quality of social relationships, restricted interests, and sex-dependent cognitive styles (1–6). Notably, many of the brain regions that are recruited for the behavioral and cognitive skills that are often impaired in individuals with ASD are subject to the organizational effects of testosterone during these critical periods of fetal development (7–9).

While androgens can be readily measured in amniotic fluid during pregnancy, several important caveats must be considered. Many studies using this approach have found that testosterone levels in amniotic fluid from male-carrying pregnancies are higher compared to levels collected from female-carrying pregnancies (10–13). However, these differences in hormone concentrations are not robust enough to reliably predict fetal sex, which may be due to the considerable overlap in average amniotic fluid hormone levels between the two sexes (4, 10, 13–16). Further, direct measurements of hormone concentrations in fetal blood across the prenatal period reveal that the observed sex difference may be specific to certain gestational time points (15, 17, 18). It must also be noted that the collection of amniotic fluid obtained during amniocentesis, an invasive medical procedure, is limited to high-risk pregnancies, including pregnancies that may be affected by conditions known to be associated with child neurodevelopment. Finally, the testosterone measured in amniotic fluid has long been thought to be of fetal origin based on the hypothesis that maternal testosterone is converted to estradiol by the aromatase (*CYP19A1)* enzyme, which is highly expressed in the human placenta (19–22). We recently reported that higher levels of maternal testosterone sampled during the second trimester of pregnancy are associated with higher scores on an ASD screener questionnaire, especially in the context of low placental aromatase. However, this system has not been thoroughly investigated especially in high-risk pregnancies, such as those affected by hypertensive disorders and polycystic ovary syndrome (PCOS).

The relationship between elevated prenatal maternal testosterone and child neurodevelopmental outcomes is further supported by the increased risk for ASD among children born to women with clinical conditions associated with hyperandrogenism, such as preeclampsia and other hypertensive disorders of pregnancy (23–29). Women with preeclampsia have significantly higher testosterone levels in the third trimester, but little is known about androgen levels at earlier time points (23, 30–32). Beyond the elevated steroidogenic activity, differences in placenta aromatase activity have been observed in preeclamptic pregnancies (24, 33, 34).

In addition to hypertensive disorders of pregnancy, increased maternal testosterone is often observed in individuals diagnosed with PCOS. PCOS is an endocrine condition that affects approximately 5-10% of women and is characterized by ovulatory dysfunction, polycystic ovaries, infertility, and hyperandrogenism (35–37). During pregnancy, circulating testosterone levels are higher and rise throughout gestation among women with PCOS (36–39). Women with PCOS are at increased risk for preeclampsia as well, though it remains unclear whether PCOS-related hyperandrogenism contributes to this (30, 38). As with hypertensive disorders of pregnancy, many studies have reported an increased risk for neurodevelopmental disorders in the children of women with PCOS (35, 37, 40–43). Two large population-based matched case-control analyses found that the odds of an ASD diagnosis were up to 60% higher in children whose mothers had with PCOS compared to healthy controls (35, 40).

While it is unlikely that these conditions directly cause ASD, they serve as a biological indication of abnormal hormone activity, which may affect fetal brain development and neuropsychiatric outcome. For example, women with PCOS are more likely to be diagnosed with psychiatric conditions, metabolic and cardiovascular conditions, and to require assisted reproductive technologies or hormonal therapy and these factors partly mediate the association between maternal PCOS and ASD risk (44). Therefore, we caution against inferring a direction of causality and rather, we suggest that the observed association between these maternal conditions and neurodevelopmental outcome in the children may inform research aimed at identifying underlying mechanisms and early emerging biomarkers.

In the current study, we sought to investigate whether the elevated maternal testosterone typical of hyperandrogenic disorders (HDs) during pregnancy, including hypertensive disorders of pregnancy and/or PCOS, could account for the increased risk for atypical child communication and social development using a matched case-control study design. Few studies have sought to identify a biological mechanism underlying the association between these conditions and risk for ASD. Given the association between maternal testosterone levels and ASD-related behaviors, the association between HDs during pregnancy and risk for ASD in children, and the hyperandrogenism often observed in the HDs included in this study, we hypothesized that maternal testosterone would mediate the relationship between HDs and ASD risk.

## Methods and materials

### Participants

This analysis was conducted as part of an ancillary study of the Nulliparous Pregnancy Outcomes Study: Monitoring Mothers-to-be (nuMoM2b) (ClinicalTrials.Gov:NCT01322529) (45). In brief, the nuMoM2b study was established in 2010 as a multi-site prospective cohort study to identify and evaluate factors that could be used to predict adverse pregnancy outcomes in nulliparous women. To participate in the nuMoM2b study, nulliparous pregnant women were screened for eligibility during the first trimester based on previously described inclusion and exclusion criteria (45).

**Figure 1:**
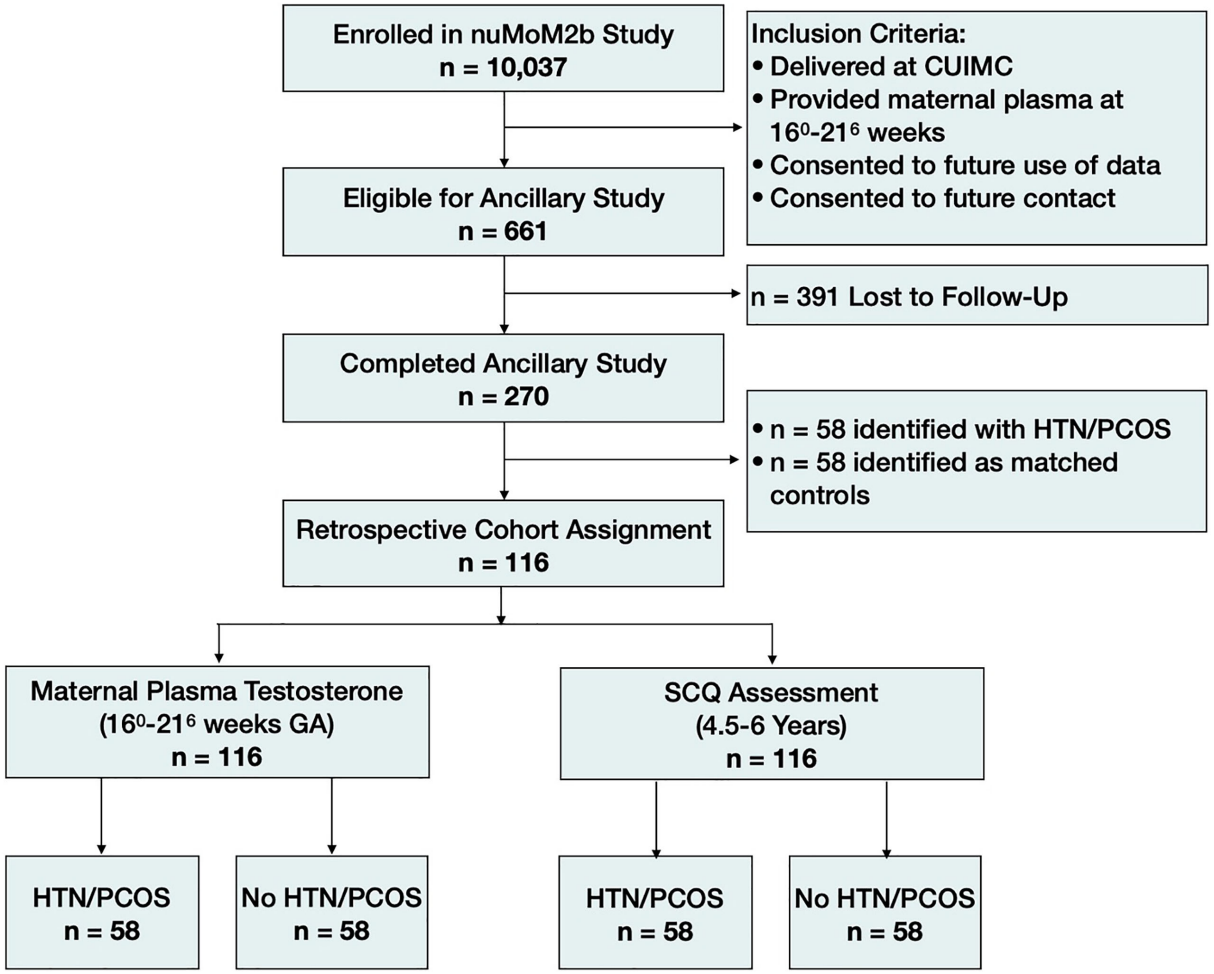
Cohort diagram. HTN/PCOS, Hypertensive disorder of pregnancy and/or polycystic ovary syndrome; SCQ, Social Communication Questionnaire.

Of the 10,037 women who enrolled in the study, 1,005 delivered at Columbia University Irving Medical Center (CUIMC). Women were eligible to participate in this ancillary study, which aimed to identify pregnancy-related determinants of neurodevelopmental risk in the children, if they participated in the original nuMoM2b study while pregnant, gave birth to a live-born infant at the CUIMC site, were fluent in English or Spanish, and were ≥ 18 years old at the time of enrollment in the nuMoM2b Study. Additionally, eligible women must have provided a sample of maternal blood during the second trimester as part of the nuMoM2b Study and consent to future use of their data and future contact by study staff. In total, 661 of the nuMoM2b study participants met criteria to participate in this ancillary study. Study staff attempted to contact all eligible mothers when their children were between 4.5-6.5 years of age to invite them to complete a parent-report neurodevelopmental assessment. Of the 661 eligible mothers, we successfully obtained child neurodevelopmental data from 270. The health status of the mother was not known at the time of data collection for this ancillary study, therefore, we implemented a retrospective cohort study design and identified 58 mothers who had a diagnosis of a hypertensive disorder of pregnancy and/or PCOS (HDs) and matched them with healthy controls, for a total of 116 participants (**Figure 1**). All procedures were reviewed and approved by the CUIMC Institutional Review Board and participants provided informed consent prior to all study procedures.

### Retrospective cohort study design

Of the 270 children for whom samples and neurodevelopmental outcome data were available, 58 were born to women who were diagnosed with a hypertensive disorder of pregnancy and/or PCOS (n=35 with a hypertensive disorder only; n=18 with PCOS only; n=5 with both a hypertensive disorder and PCOS). From the remaining 212 children, we retrospectively identified 58 frequency-matched controls based on fetal sex. To avoid biases in age at follow-up or other possible confounding factors related to data collection, potential controls were assigned a randomized numerical identifier and re-ordered accordingly. Following numerical order, we matched one control to each case based on the children’s sex (male versus female) while remaining blind to all other data.

### Identifying cases of pregnancy-related hypertension and/or PCOS

Study staff reviewed electronic medical records to ascertain the provider’s primary reason for delivery and whether the participant had demonstrated hypertension or proteinuria during the pregnancy. Participants for whom the primary reason for delivery was 1) gestational hypertension, 2) mild preeclampsia, 3) severe preeclampsia, 4) unspecified preeclampsia, 5) eclampsia, 6) HELLP syndrome, or 7) incomplete HELLP syndrome were classified as having a hypertensive disorder of pregnancy. Additionally, participants were classified as having hypertensive disorder of pregnancy if they had demonstrated hypertension or proteinuria during their pregnancy. Participants were asked to self-report whether they had received a diagnosis of PCOS as part of the online follow-up survey, and whenever possible, diagnoses were confirmed through the electronic medical record.

### Child neurodevelopmental assessment

When children reached 4.5-6.5 years of age (mean = 62.1 months, range = 54.0-79.0 months), mothers were invited to complete the Social Communication Questionnaire (SCQ) through a secure online portal. The SCQ (lifetime version) is a parent-report questionnaire developed to detect ASD-related behaviors in children beginning at four years of age (or mental age of two years) (46). The SCQ was developed to parallel the Autism Diagnostic Interview Revised (ADI-R), one of the “gold standards” for diagnosing ASD in children and has established validity with the ADI-R with robust agreement in identifying autism. The SCQ contains 40 ‘yes’ or ‘no’ items that pertain to behavioral domains that are often disrupted in children with ASD (e.g., verbal and non-verbal communication, reciprocal social interaction, restricted or repetitive behaviors). Based on these 40 items, a total score ranging from 0-40 is calculated, with higher scores indicating more ASD-related behaviors, including deficits in communication skills and social functioning. When administered for children 4 years of age and older, the SCQ has a sensitivity of 0.56 and a specificity of 0.74 (47).

#### Maternal plasma in the second trimester

Whole blood was collected from participating women between 16^0^-21^6^ weeks gestational age by venipuncture into an EDTA-coated tube. The whole blood was centrifuged at 1500g for 10 minutes at 4°C and plasma was extracted and stored at -80°C until further processing.

### Testosterone radioimmunoassay in second trimester maternal plasma

To measure total testosterone in maternal plasma samples collected in the second trimester, a radioimmunoassay was performed using commercially available kits and conducted following guidelines provided by the supplier (MP Biomedicals, Cat# 07-189105). All samples were run in duplicate, and values were averaged. The intra-assay coefficient of variation (CV) was 11.15% and the lower limit of detectability (LLD) was 0.09 ng/ml. Three samples that were below detection (e.g., <0.09) were assigned the value of the lowest level of detectability.

### Statistical analyses

Statistical analyses were conducted using R and SPSS software. Total SCQ scores and maternal testosterone values were positively skewed and 1-minute Apgar scores, neonatal head circumference, and infant gestational age were negatively skewed and were square root transformed prior to statistical analysis. For all analyses, women with a hypertensive disorder of pregnancy and/or PCOS were assigned to the exposure group (HD group) and women without a diagnosis of either condition were assigned to the control group. Welch’s two sample two-tailed t-tests were performed to determine whether women diagnosed with an HD differed from the matched controls with regard to maternal testosterone levels and total SCQ scores. These tests were repeated after stratifying the data by sex (males versus females). We examined several covariates relating to parental characteristics, the course of the pregnancy, and neonatal outcomes that were identified *a priori* as potential confounding risk factors, including maternal and paternal age at delivery, maternal education, maternal self-reported ethnicity, mode of delivery, gestational age at birth, birth weight, 1-minute Apgar scores, neonatal head circumference, and the child’s age at the time of follow-up. Bivariate correlations between continuous covariates and the primary dependent and independent variables were conducted. We also performed Welch’s two sample t-tests and a chi-square tests of independence to determine whether any of our covariates of interest differed between the cases and frequency-matched controls. Candidate covariates were included in the adjusted linear models if they were associated with either the dependent or independent variables at a significance level of 0.1. Linear regression models were performed after sex stratification and were found to be normal. Finally, we used the ‘mediation’ package in R to perform a mediation analysis using bootstrapping methods to determine the mediating effects of maternal testosterone on the relationship between a maternal HD and female children’s neurodevelopmental outcome. A statistical power analysis was performed for sample size estimation and revealed that with an alpha = .05 and power = 0.80, the projected sample size needed is approximately n = 53 in each study group (n = 106 in total) for the between group comparison. Therefore, our sample size of n= 58 in each group (n = 116 in total) is sufficient.

## Results

### Sample characteristics

Sample characteristics were provided by the nuMoM2b data coordinating and analysis center and are shown in **Table 1**. The two groups did not differ significantly with regard to any other clinical or demographic characteristics, including maternal and paternal age at delivery, gestational age at birth, birth weight, neonatal head circumference, 1-minute Apgar scores, mode of delivery, maternal and paternal ethnicity, and maternal education. Children were case-matched based on sex, resulting in 32 males and 26 females in each exposure group. The child’s age at assessment did not differ between the two groups.

**Table 1:** Study sample characteristics.

Characteristic	No HTN/PCOS	HTN/PCOS	χ2 or *t* statistic	*p* value
	Mean± SD (*n*)	Mean± SD (*n*)		
**Maternal age (years)**	29.2±5.8 (58)	29.1±5.6 (58)	0.13	0.90
**Paternal age (years)**	30.9±6.7 (55)	31.3±7.7 (57)	-0.27	0.79
**Gestational age at birth (days)**	276.2±8.8 (58)	272.7±12.7 (58)	1.72	0.09
**Birth weight (grams)**	3254±376 (58)	3176±515 (58)	0.94	0.35
**Infant head circumference (cm)**	33.9±1.2 (56)	33.8±1.7 (54)	0.52	0.60
**1-minute Apgar score**	8.5±0.98 (58)	8.2±1.2 (58)	1.42	0.16
**Child age at follow-up (months)**	62.6±5.9 (58)	62.7±5.2 (58)	0.87	0.39
	***% (n*)**	***% (n*)**		
**Infant sex**			0.00	1.0
** Male**	55 (32)	55 (32)		
** Female**	44 (26)	44 (26)		
**Mode of delivery**			0.04	0.84
** Vaginal**	71 (41)	69 (40)		
** Cesarean**	29 (17)	31 (18)		
**Maternal Ethnicity**			2.79	0.10
** Hispanic**	43 (25)	58 (34)		
** Non-Hispanic**	57 (33)	41 (24)		
**Paternal Ethnicity**			0.22	0.64
** Hispanic**	47 (27)	50 (29)		
** Non-Hispanic**	53 (31)	50 (28)		
**Maternal Education**			0.61	0.96
** No high school degree**	2 (1)	2 (1)		
** High school degree**	9 (5)	7 (4)		
** Some college, no degree**	15 (9)	19 (11)		
** College degree**	33 (19)	36 (21)		
** Graduate/professional degree**	41 (24)	36 (21)		

HTN/PCOS, Hypertensive disorder of pregnancy and/or polycystic ovary syndrome.

### Differences in maternal testosterone by exposure group

**Figure 2:**
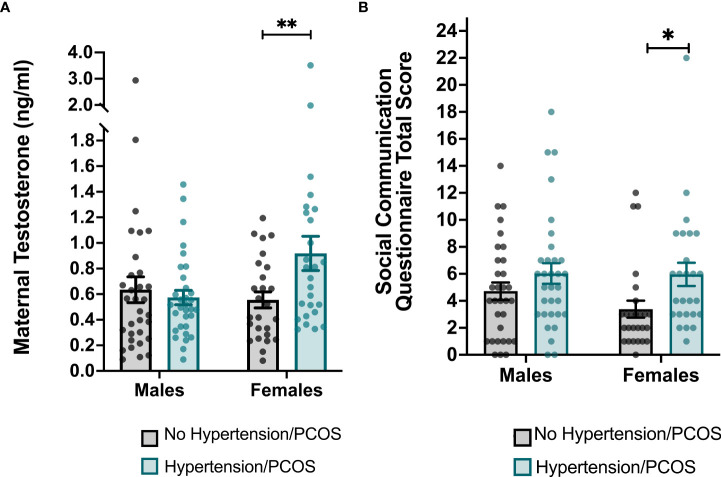
Group differences in maternal testosterone levels and total scores on the Social Communication Questionnaire (SCQ) stratified by sex. **(A)** Maternal testosterone levels were significantly higher in women who were diagnosed with a hypertensive disorder of pregnancy and/or PCOS and who carried a female. **(B)** Total scores on the SCQ were significantly higher among female children born to women with a hypertensive disorder of pregnancy and/or PCOS. Each point represents an individual subject. Error bars represent the SEM. *p < 0.05, **p ≤ 0.01.

There were no group differences in second trimester maternal testosterone levels between women with an HD during pregnancy and matched controls (*p* = 0.09). However, among women carrying a female fetus, maternal testosterone levels in the second trimester were significantly higher in women with an HD (0.91±0.30) compared to the matched controls (0.71±0.22) (*t* (48) = -2.69, *p* = 0.01) (**Figure 2A**).

### Differences in child neurodevelopmental by exposure group

We performed Welch’s two sample two-tailed t-tests to determine whether ASD-related behaviors as measured by total SCQ scores differed between children exposed to an HD and matched controls. Compared to healthy controls, children born to mothers with an HD were reported to have significantly greater ASD-related behaviors as indicated by higher total SCQ scores (cases: 2.29±0.88; controls: 1.82±0.91; *t (*114) = -2.82, *p* =0.01). After stratifying data by infant sex, the relationship between maternal HDs during pregnancy and total SCQ scores remained significant among females (HD cases: 2.31±0.80; controls: 1.67±0.79; *t (* 48) = -2.92, *p* =0.01), but not males (**Table 2** and **Figure 2B**). After performing an adjusted linear regression that controlled for covariates that were associated with either the independent or dependent variables, the diagnosis of an HD remained a significant predictor of total SCQ scores in females (*p* = 0.03).

**Table 2:** Group differences on Social Communication Questionnaire stratified by sex.

	Females					Males				
	No HTN/PCOS	HTN/PCOS				No HTN/PCOS	HTN/PCOS			
	Mean±SD	Mean±SD	*df*	*t*	*p*	Mean±SD	Mean±SD	*df*	*t*	*p*
**SCQ Total Score^†^**	1.67±0.79	2.31±0.80	50	-2.92	**0.01**	1.94±0.98	2.27±0.95	62	-1.34	0.19

^†^Square-root transformed. HTN/PCOS, Hypertensive disorder of pregnancy and/or polycystic ovary syndrome; SCQ, Social Communication Questionnaire. Bold values indicate a statistically significant p-value.

### Mediating effects of maternal testosterone on the association between prenatal maternal conditions and total SCQ scores

The effect of a hypertensive disorder of pregnancy and/or PCOS on ASD-related behaviors in females was partially mediated by maternal testosterone during pregnancy. The standardized regression coefficient between the diagnosis of a maternal HD and total SCQ scores is statistically significant when controlling for covariates that are associated with both independent and dependent variables (*p* = 0.03). The standardized regression coefficient between maternal testosterone levels and ASD-related behaviors as indicated by total SCQ scores was significant (*p* = 0.02), though the regression was not significant when controlling for the mother’s HD status (*p* = 0.11). The standardized indirect effect was (.20)*(.67) = .13. We tested the significance of this indirect effect using bootstrapping procedures. Unstandardized indirect effects were computed for each of 1,000 bootstrapped samples, and the 95% confidence interval was computed by determining the indirect effects at the 2.5th and 97.5th percentiles. The bootstrapped unstandardized indirect effect was .13, and the 95% confidence interval ranged from 0.00, 0.31 (*p* = 0.06).

## Discussion

This analysis replicated and expanded previous findings. Our results demonstrate that maternal testosterone levels in the second trimester are significantly higher in women diagnosed with an HD, but only among women carrying a female fetus. Similarly, parent-reported ASD-related behaviors were significantly higher in female, but not male children born to women with an HD. Consistent with our hypothesis, we found that maternal testosterone levels in the second trimester partially mediate the association between HDs during pregnancy and child neurodevelopment.

It has been well-documented that hypertension during pregnancy is associated with increased risk for ASD in the offspring, but few studies have directly evaluated the biological pathways underlying this association using *in vivo* methods in human subjects. Several biological mechanisms have been proposed, including abnormal differentiation of trophoblast cells, fetal hypoxia and oxidative stress, and exposure to a heightened maternal inflammatory state (27, 29). To our knowledge, hyperandrogenism in pregnant women with hypertension has not previously been studied as a potential mechanism through which hypertension during pregnancy could impact the fetal brain and increase risk for neurodevelopmental disorders. In contrast, it has been widely suggested that elevated testosterone levels in women with PCOS may be associated with greater fetal exposure to testosterone, which could account for the increased prevalence of ASD in children born to women with PCOS (35, 41, 42, 49). In fact, some studies have used maternal PCOS as a proxy for fetal testosterone exposure to study the effects on neurodevelopmental outcome (40).

Here, we have expanded on the previously reported finding that maternal testosterone levels are higher in women diagnosed with hypertensive disorders of pregnancy and PCOS (23, 30–32) by demonstrating this effect to be sex-specific and only present in female-carrying pregnancies. One previous study reported higher maternal testosterone in women with preeclampsia who had male fetuses, but testosterone was sampled during the third trimester, whereas we sampled testosterone in the second trimester (48). Therefore, it is possible that the timing of collection may bias the sex-specific effects.

Our results also support the previously observed independent associations between hypertensive disorders of pregnancy and PCOS and child neurodevelopmental outcomes (25–29, 35, 37, 40–43). Different from prior studies, we combined pregnancies affected by hypertension and/or PCOS into one exposure group. One study found a moderate compounding effect of maternal PCOS and maternal metabolic conditions on ASD risk, though only the effect of obesity was statistically evaluated (35). We combined the two conditions based on our hypothesis that the associations between each condition and neurodevelopmental risk were mediated by the hyperandrogenism that is characteristic of both conditions. We found that female children born to mothers with a hypertensive disorder of pregnancy and/or PCOS had higher total SCQ scores after adjusting for several important covariates. To our knowledge, a sex-dependent effect of hypertensive disorders of pregnancy on child communication and social skills has not been previously reported. Despite strong evidence that hypertensive disorders of pregnancy are associated with increased risk for ASD, prior studies have either controlled for fetal sex as a covariate or have not addressed it as a variable of interest (25–29). In the case of PCOS, contrasting sex-specific effects have been reported. In some cases, studies have shown that daughters of women with PCOS are at increased risk for ASD, while others have reported increased risk among sons born to women with PCOS (35, 37, 40). Here, we present evidence of a sex-specific effect of hypertension and/or PCOS such that only exposed daughters exhibit greater ASD-related behaviors. Fetal exposure to maternal hyperandrogenism may impact long-term behavioral outcomes through ‘organizational effects,” which occur during critical periods of brain development and plasticity when hormone-dependent neural systems are being established (2, 7, 50). Many of the first brain regions to express androgen receptors are those that are involved in regulating sexually dimorphic behaviors, such as the medial preoptic area (MPOA) within the hypothalamus and the spinal nucleus of the bulbocavernosus (SNB) (7, 9). Notably, many of the brain regions that are recruited for the behavioral and cognitive skills that are often impaired in individuals with ASD are also subject to the organizational effects of testosterone during these critical periods of fetal development (7–9). Therefore, prenatal exposure to maternal hyperandrogenism may shape hormone-dependent brain regions that have been implicated in the etiology of neurodevelopmental disorders, such as ASD.

This study has several strengths, including the retrospective cohort study design, which allowed us to match each HD case to a sex-matched control. The nuMoM2b Study was established to identify gestational risk factors among first-time mothers, and therefore, the sample is comprised solely of nulliparous women. Many previous studies include both nulliparous and multiparous pregnancies, which may confound the pathophysiology of the reported associations between maternal HDs and child neurodevelopmental outcomes. Finally, the nuMoM2b study sample enrolled through the CUIMC site is demographically diverse. In addition to these strengths, we acknowledge several limitations. The majority of children in this sample did not meet the established cut-off score of 10 for a clinical ASD evaluation and the SCQ is not a diagnostic instrument (47). Therefore, increased SCQ scores may indicate subclinical ASD-related behaviors, but the associations presented here may not be applicable to children with a formal ASD diagnosis. To illustrate this point, we further examined the six children in our sample who were reported to have a diagnosis of ASD at the time of the assessment. Five of the six children were males born to mothers with a hypertensive disorder of pregnancy and/or PCOS; two of the males were born to mothers with a hypertensive disorder of pregnancy, two were born to mothers with PCOS, and one was born to a mother with both hypertension and PCOS. Our data suggest that these conditions are more strongly associated with ASD-related behaviors in female children, though these effects may only be true for subclinical behaviors. Alternatively, it has been well-documented that females with ASD are better able to mask their symptoms and therefore, undiagnosed females may be a particularly vulnerable population that is more likely to go undiagnosed (51). It is also important to note that we measured total testosterone, which includes both bound and free testosterone. Since we did not include a measure of sex hormone-binding globulin (SHBG), which is known to increase during pregnancy (52), we cannot independently determine the influence of free testosterone. Identification of women with PCOS was largely dependent on self-reporting and subsequent confirmation through electronic health records. However, self-reported diagnoses present the possibility of inaccuracy and further, specific criteria used to make the clinical diagnosis were not available in this dataset. Finally, the number of participants included in the matched case-control analysis is small and thus, statistical power may be limited.

The association between elevated maternal testosterone in the second trimester and risk for ASD-related behaviors in their children has important implications for early diagnosis and management of ASD. Female children with ASD often go undiagnosed or experience delays in diagnosis, but the sex-specific associations between hypertensive disorders of pregnancy/PCOS, maternal hyperandrogenism, and increased ASD-related behaviors in female children provides a potential early marker for risk among females, specifically. Thus, maternal testosterone should be further investigated as a potential early, noninvasive biomarker to identify subsets of children who are at risk for developing ASD. Ultimately, early emerging biomarkers may allow for earlier diagnosis and intervention, which could improve functioning and overall quality of life of affected individuals.

## Data availability statement

The datasets presented in this study can be found in online repositories. The names of the repository/repositories and accession number(s) can be found below: https://dash.nichd.nih.gov/study/226675.

## Ethics statement

The studies involving human participants were reviewed and approved by Columbia University Institutional Review Board. The patients/participants provided their written informed consent to participate in this study.

## Author contributions

MF, WG, DH, SP, UR, RS, RW, and FC contributed to conception and design of the study. MF, RR, HW, and DG generated and organized the database. MF performed the statistical analysis. MF wrote the first draft of the manuscript. FC wrote sections of the manuscript. All authors contributed to the article and approved the submitted version.

## Funding

This research was supported by Award Number P2CHD058486 awarded to the Columbia Population Research Center from the Eunice Kennedy Shriver National Institute of Child Health & Human Development.

## Acknowledgments

We thank the institutions and researchers involved in the Nulliparous Pregnancy Outcomes Study: Monitoring Mothers-to-be (nuMoM2b) Network, the nuMoM2b Steering Committee, Michelle DiVito, and Caroline Torres for providing the necessary data and logistical support throughout the project. We also thank the families who generously contributed to this work.

## Conflict of interest

The authors declare that the research was conducted in the absence of any commercial or financial relationships that could be construed as a potential conflict of interest.

## Publisher’s note

All claims expressed in this article are solely those of the authors and do not necessarily represent those of their affiliated organizations, or those of the publisher, the editors and the reviewers. Any product that may be evaluated in this article, or claim that may be made by its manufacturer, is not guaranteed or endorsed by the publisher.

## Abbreviations

ASD, autism spectrum disorder; HD, hyperandrogenic disorder; nuMoM2b, Nulliparous Pregnancy Outcomes Study: Monitoring Mothers-to-be; PCOS, Polycystic ovary syndrome; CUIMC, Columbia University Irving Medical Center; SCQ, Social Communication Questionnaire.
